# News and Coming Events

**Published:** 1908-01-25

**Authors:** 


### NEWS AND COMING EVENTS.

Dr. William Hunter has been appointed to the post of physician to Charing Cross Hospital, vacant through the death of Dr. Montague Murray.

The annual general meeting of the governors of the Italian Hospital will be held at the hospital, Queen Square, London, W.C., on Saturday, January 25, 1908, at 4 p.m.

The reconstructed block for female patients at the London Fever Hospital, Liverpool Road, W., will be opened by Lady Balfour of Burleigh on Monday next, the 27th inst., at 3 p.m.

The Royal National Orthopædic Hospital has received a contribution of £262 7s. 11d. from the trustees of Smith's Kensington Estate Charity; and also the notice of a legacy of £200 under the will of the late Richard Boyle.

A meeting will be held at the Medical Graduates' College and Polyclinic, 22 Chenies Street, Gower Street, London, on Monday, the 27th inst., at 8 o'clock, to consider the proposal to form a British Ship-Surgeons' Association.

The West London Hospital, Hammersmith, W., has received from the executors an award of £200 (less 10 per cent. legacy duty) out of the charitable bequest of the late Mr. J. L. Toole, and a donation of £52 10s. from the Salters' Company.

The Earl Cawdor, as Treasurer of the London Homœopathic Hospital, Great Ormond Street, W.C., has received the sum of £2,000 from Lord Dysart in fulfilment of his promise towards the £30,000 required for the extension of the hospital on its own freehold site.

The Therapeutical and Pharmacological Section of the Royal Society of Medicine will meet at the Apothecaries' Hall, Blackfriars, E.C., on Tuesday, January 28, at 4.30 p.m. Professor W. E. Dixon will read a paper on "Arterio-sclerosis and its Causation," and Professor A. R. Cushing one on "Nutmeg Poisoning."

Royal Society of Medicine (Odontological Section).—The next meeting of this section will be held on Monday next, January 27, at 8 p.m., when the Curator will show some recent additions to the museum. Mr. W. W. James, F.R.C.S., L.D.S., will read some notes on a case of extensive necrosis of the mandible (the patient will be in attendance before the meeting). Mr. J. F. Colyer will read a paper on the treatment of children from a dental aspect (illustrated by the epidiascope).

At the quarterly meeting of the council of the Royal College of Surgeons of England, held on Thursday, January 16, the revised standing rules relating to the office of conservator of the museum were approved, and it was determined to proceed to the election of a conservator in the vacancy occasioned by the death of Professor Stewart, at the next meeting of the council on February 13. Mr. Henry Morris was appointed Hunterian Orator for the year 1909, and Mr. Thomas Bryant was re-elected the representative of the college upon the council of the Queen Victoria's Jubilee Institute for Nurses. A committee was appointed to prepare a circular to the fellows and members upon the question as to whether women should be admitted to examination for the diplomas of the college. A suggestion that arrangements should be made for serving tea to readers in the library was adopted.

Mr. W. McAdam Eccles, F.R.C.S., will deliver the Lees and Raper Memorial Lecture in the Town Hall, Oxford, on February 4 next at 8 p.m.

The Royal National Orthopædic Hospital has received a contribution of £52 10s. towards the general funds of the Hospital, from Mrs. James Packe.

In an action for libel brought on behalf of the proprietors of an asthma specific against the *Lancet*, a King's Bench Jury has awarded the plaintiff damages to the amount of £1,000. A stay of execution was granted on the usual terms.

The Right Hon. the Earl of Plymouth, P.C., C.B., D.L., J.P., Lord Lieutenant of Glamorganshire, will preside over the twenty-fourth annual Congress and Exhibition of the Royal Sanitary Institute at Cardiff from July 13 to 22, 1903. The public meeting to inaugurate arrangements for the Congress will be held at the City Hall, Cardiff, on Thursday, February 6.

On Friday, January 31, a lecture will be delivered before the Child Study Society, London, Parkes Museum, Margaret Street, W., on "Physical Activities as a Basis for Education in Home and School," by Professor W. A. Baldwin, Principal of the State Normal School, Hyannis, Mass., U.S.A. (who is on a short visit to this country). The chair will be taken at 8 p.m. by C. W. Kimmins, M.A., D.Sc., Chief Inspector Education London County Council.

The Society of Medical Phonographers.—We have been asked to inform any members of the profession or students who use Phonetic shorthand that this Society still exists and still issues its medical periodical in lithographed phonography to promote the more effective use of shorthand in note-taking. The President is again Sir William Gowers, who was the chief agent in its foundation twelve years ago. Dr. Sims Woodhead and Mr. Cathcart, of Edinburgh, are the Vice-Presidents. The Hon. Sec., who will be glad to give particulars regarding the Society, is Dr. Gruner, Pathological Laboratory, General Infirmary, Leeds.

The newly-created post of Medical Superintendent at St. George's Hospital has been filled by the appointment of Mr. G. E. Friend, M.R.C.S., L.R.C.P., who is at present holding the post of assistant pathologist. Mr. Friend has also been house surgeon and house physician at St. George's, as well as acting resident medical officer; and house surgeon and house physician also to the Victoria Hospital for Children. To those who have been familiar with the extremely successful and tactful way in which he has carried out the duties of those posts, Mr. Friend's appointment will occasion no surprise; in his hands the success of the new experiment at Hyde Park Corner should be assured.

Dr. Arthur Newsholme, Medical Officer of Health for Brighton, has been appointed chief medical officer to the Local Government Board in succession to Dr. W. H. Power, C.B., resigned. Dr. Newsholme is well known for his writings on hygiene and public health, and has been president of the Incorporated Society of Medical Officers of Health. The appointment has drawn the attention of an influential section of the lay press to the suggestion, which has been frequently discussed in these columns, for the constitution of a Ministry of Public Health. Dr. Newsholme is a graduate of London University and received his medical education at St. Thomas's Hospital.

